# Study Progress of Radiomics With Machine Learning for Precision Medicine in Bladder Cancer Management

**DOI:** 10.3389/fonc.2019.01296

**Published:** 2019-11-28

**Authors:** Lingling Ge, Yuntian Chen, Chunyi Yan, Pan Zhao, Peng Zhang, Runa A, Jiaming Liu

**Affiliations:** ^1^West China Hospital, Sichuan University, Chengdu, China; ^2^Radiological Department, West China Hospital, Sichuan University, Chengdu, China; ^3^Department of Urology, Institute of Urology, West China Hospital, Sichuan University, Chengdu, China; ^4^Department of Obstetrics and Gynecology, West China Second Hospital, Sichuan University, Chengdu, China

**Keywords:** radiomics, machine learning, bladder cancer, full-cycle management, precision medicine

## Abstract

Bladder cancer is a fatal cancer that happens in the genitourinary tract with quite high morbidity and mortality annually. The high level of recurrence rate ranging from 50 to 80% makes bladder cancer one of the most challenging and costly diseases to manage. Faced with various problems in existing methods, a recently emerging concept for the measurement of imaging biomarkers and extraction of quantitative features called “radiomics” shows great potential in the application of detection, grading, and follow-up management of bladder cancer. Furthermore, machine-learning (ML) algorithms on the basis of “big data” are fueling the powers of radiomics for bladder cancer monitoring in the era of precision medicine. Currently, the usefulness of the novel combination of radiomics and ML has been demonstrated by a large number of successful cases. It possesses outstanding strengths including non-invasiveness, low cost, and high efficiency, which may serve as a revolution to tumor assessment and emancipate workforce. However, for the extensive clinical application in the future, more efforts should be made to break down the limitations caused by technology deficiencies, inherent problems during the process of radiomic analysis, as well as the quality of present studies.

## Introduction

Bladder cancer ranks ninth of the most common malignancies and the 13th most common predisposing cause of cancer-related mortality all over the world, with over 357,000 new cases and over 130,000 deaths annually (1). Bladder cancer is more likely to develop in patients over 65 years old, which has a high recurrence rate ranging from 50 to 80% (2).

Clinical decision and follow-up management of bladder cancer predominantly depend on the presence or absence of muscle invasion and accurate grade of malignancy and also take specific pathological types into consideration (3, 4).

However, all aspects in the management of bladder cancer including tumor staging, diagnosis, treatment, and prognostic evaluation have still been limited by various factors. The gold standard nowadays for bladder cancer detection is telescopic checking of the bladder (cystoscopy) (5). Considering the high recurrence rate, cystoscopy examinations are required to be performed every 3–6 months to monitor bladder cancer patients for recurrence or progression to a more advanced stage. However, the expensive and invasive characteristics restrict the frequent use of cystoscopy and further cause significant economic and psychological pressure to patients. Furthermore, cystoscopy has still quite limited accuracy for the detection of tumors in low grade, with the sensitivity of 61% (6), nor the muscle-invasive depth. Another universally applied approach in condition detection is biopsy but it can be restricted by an inability to sample every part of the tumor at any point in time. Moreover, heterogeneous disease spectrums as bladder cancer processes, it can always confound correct classification and staging (5), and then influences the choice of treatment plans and finally makes the risk of undertreatment or overtreatment increase. To simplify the detection process of bladder cancer, the concept of urinary biomarkers has been put forward recently. However, no studies have proposed molecular markers with sufficient sensitivity and specificity to replace cystoscopy (1). Furthermore, this method is unable to determine the extent of surrounding tissue invasion and metastasis, which blocks the way of non-invasive bladder cancer detection.

Apart from the difficulties in accurate tumor detection and clinical grading, the prediction of treatment efficacy is also a major obstacle in the management of bladder cancer. It has been widely accepted that patients with nonmuscle-invasive bladder cancer (NMIBC, stage ≤T1) are mostly at early stage and are advised to be treated with TURBT followed by treatment applying Bacillus Calmette-Guérin (BCG) (7), whereas muscle-invasive bladder cancer (MIBC, stage ≥T2) patients usually have a poorer prognosis and the treatment plan for these patients is supposed to be radical cystectomy (RC) (8). Despite appropriate cancer control in local lesions, over 50% of patients who have undergone RC meet the disturbance of tumor metastasis in no more than 2 years after cystectomy and thus fail to survive (9). Neoadjuvant chemotherapy ahead of cystectomy has been demonstrated to reducing the odds of developing extravesical lesions when compared to taking RC alone, afterward improves the overall survival (OS) of bladder cancer patients (10, 11). However, there is still short of a reliable method to predict the posttreatment response of a specific individual to whether BCG or neoadjuvant chemotherapy currently. Thus, some patients who receive inappropriate therapies tend to suffer from adverse reactions. Worse, these patients are likely to miss the best time to make an adjustment on the strategies of therapy, consequently pose damage to their physical condition and increase the difficulty of cancer management.

Confronting the above problems and limitations, a novel concept of radiomics has emerged for solving the issues of the generalization of precision medicine and how it can be applied in the field of bladder cancer monitoring. It is a high-throughout quantitative feature extraction method to mine the information contained in the multimodality medical images including computed tomography (CT), positron emission tomography (PET), magnetic resonance imaging (MRI), and ultrasonography (US) (12), then comprehensively analyze these massive images to extract phenotypic features (also known as radiomics biomarkers) and explore the associations between patients' prognosis and these extracted features and improve the decision-making process. ML algorithms on the basis of “big data” are fueling the powers of radiomics in three main tasks related to bladder cancer imaging: initial detection of the existence and localization of volume; pretreatment characterization including the diagnosis, grading, and staging of tumor; posttreatment monitoring by predicting prognosis or factors irrelevant to treatment plans, such as OS, recurrence, and pathological subtypes (13, 14). Therefore, radiomics methods in combination with an optimal ML method may potentially extend the practical use of precision medicine approaches in radiotherapy by providing a non-invasive, high-efficiency, but low-cost way to predict clinical outcomes (15).

In this paper, we review the present studies in association with our topic and discuss the promising usages and hidden challenges of this novel method adapting acute imaging analysis, combining radiomics with ML in the precise management of bladder cancer. This review paper considers and discusses the issues as follows:

1. The concept of radiomics and its significance
2. Workflow of radiomics
3. Clinical applications of the combination of radiomics with ML in bladder cancer management
4. Challenges and future directions.

## The Concept of Radiomics and Its Significance

The meaning of “precision medicine” indicates that the reasonable strategies of treatment are singled out according to the characteristics of different subtypes. It has substantially changed the treatment strategies in the recent 10 years. To make a precise treatment for an individual, accurate detection, characterization, and monitoring after treatment are very important. Unfortunately, the current tumor assessment is far from our expectation because of variable technology deficiency. One important problem is that radiologists usually use subjective, qualitative features to make tumor assessment, which make the results less reproducible and more unstable. Besides, with the rapid development of gene therapy and immunotherapy, gene expression signature and immune phenotype are also essential parts for a comprehensive tumor assessment. Current evaluation for gene expression and immune phenotype is most based on the biopsy, which is invasive and expensive, let alone the result is confused because of intratumoral heterogeneity. Thus, the demand for a non-invasive, cheap, and stable method to assess and monitor tumor has never been greater.

The computational medical imaging, also known as radiomics, was first invented by Lambin in 2012 (16, 17). It was based on the underlying hypothesis that medical imaging contains much more information than we have already utilized, even including cellular and molecular information of target tissue (18). The aim of radiomics is to analyze and translate medical images into quantitative data and provide an image-based biomarker to aid clinical decisions. Compared with biopsy, radiomics biomarker is invasive, reproducible, and has the ability to make an evaluation of tumors' microenvironment, spatial heterogeneity, and longitudinal assessment for disease progression. In recent years, several studies have presented the potential usage of radiomics in the development of precision medicine. Among all, many pieces of research demonstrated the immense application value of radiomics in combination with ML algorithms to overcome the drawbacks of precision medicine in the diagnosis and treatment of non-small-cell lung cancer (19). Since the breakthrough in the intelligent management of lung cancer, researches on the application of this new technology to other cancers have been carried out one after another with great advances among them. The characteristics of bladder cancer itself and the need for enhanced imaging analysis technology make it one of the major research hot spots.

## Workflow of Radiomics

**Figure 1:**
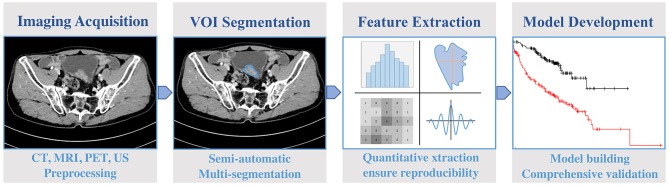
A typical workflow of radiomics in bladder cancer.

Radiomics is a multidisciplinary-based technology. There are four main steps to complete a radiomics program (Figure 1):

(1) Image acquisition and preprocessing
(2) Volumes of interest (VOIs) segmentation
(3) Feature extraction and quantization
(4) Model building.

### Image Acquisition

Image acquisition is the first and an important step in radiomics.

There are two common formats of the image data recorded, including Picture Archiving and Communication System (PACS) and the Digital Imaging and Communication in Medicine (DICOM), and they are used in most of the medical institutions, which provide a great convenience for the radiology study.

The accuracy and reproducibility of the final radiomics model lie on the quality of image acquisition. However, there is no guideline nor consensus on image acquisition. As a result, the acquisition strategies in different research teams can be distinctive, which can cause heterogeneity among separate studies. Meanwhile, it is difficult for researchers to take labeling, annotation, segmentation, and quality assurance seriously. Because these processes require well training, wasting both time and money.

Various imaging modalities such as modern CT, MRI, PET, and US scanners allow for acquisition and image reconstruction in wide variations (20). The radiological images applied for radiomic analysis are obtained from different hospitals or institutions using divided parameters and protocols. Thus, they are supposed to be preprocessed to ensure consistency and comparability.

### VOIs Segmentation

The region for image data capture is defined as “The Volume of Interest” (VOI). VOIs segmentation is the core step of radiomics study because it determines which volume is analyzed within a medical image.

The ideal VOI includes the complete information for the target lesions, nothing more nor less. Unfortunately, usually, it is hard for the radiologists to make it because many tumors have indistinct borders (21). Besides, the microenvironment around the lesions also provides useful information of the lesions, but there is no guideline in VOIs segmentation for how much microenvironment should be put in radiomics model. For instance, in a recent radiomics study, the research team use 2-mm peripheral ring on each side of the lesions to involve microenvironment of the lesions while another team use 1-mm peripheral ring (22).

VOIs can be created manually, automatically, or semiautomatically. In the past 5 years, most current radiomics study created VOIs manually. However, it is time-consuming and laborious when utilizing big data in radiomics study. Thus, many pieces of research try to create VOI automatically. There are lots of algorithms on VOI creation. A common segmentation algorithm is the “seed method.” The radiologist will place some seeds in VOI, and the computer will create VOI automatically (23). Unfortunately, it only works well when the lesion is uniform. There are many other methods used in radiomics study, such as Graph-Cut Methods (24), Level-Set Methods (25), and Active Contour Algorithms (26). In summary, all algorithms have their own deficiencies and need manual correction. Therefore, there is a great demand for algorithms with maximum automation, minimal human intervention, high time efficiency, and repeatability.

### Feature Extraction

Feature extraction is the next step after a VOI is reasonably segmented, which is the essence in the workflow of radiomics. It mainly performs the extraction of high-throughput data of quantitative imaging features to identify VOIs and the selection of useful information to aid in the discrimination of normal and abnormal images.

The quantitative data usually can be classified into four types:

(1) **Shape characteristics:** Description of the shape and geometric features of target lesions. For example, the volume of VOI, maximum diameter along with different orthogonal directions, maximum surface area, tumor compactness and sphericity.
(2) **First-order statistical characteristics:** Description of the distribution of individual but do not describe its spatial arrangement, including the mean, median, maximum and minimum of voxel intensity, skewness (asymmetry), kurtosis (flatness), uniformity, and randomness (entropy).
(3) **Second-order statistical characteristics:** Also known as texture features. It is calculated from a statistical correlation between adjacent voxels, usually by Gray-Level Co-Occurrence Matrix (GLCM) (27). Texture features provide heterogeneity information among the lesions.
(4) **High-order statistical characteristics:** Quantitative data from the filter or mathematical transformation on images, including fragment analysis, Minkowski function, wavelet transform, and Laplace transform of gaussian filtered image. The aim of filer and mathematical transformation is to identify repetitive or non-repetitive patterns, suppress noise, or highlight details.

### Model Building

To conclude, the building of radiomics model involves three main aspects, including the selection of radiomic features, choice of ML methods, and final validation of the developed model. Image analysis software can provide many features, so it is vital to select essential features for further study. At the same time, model selection is as important as feature selection. Selecting a suitable model can help obtain a reliable and stable result, which is very important for clinical decision making. Complete radiomics analysis should include validation, and both internal validation and external validation are indispensable.

#### Feature Selection

There are two common processes for determining radiomic parameters. One is to make a preliminary analysis of captured features and select most repeatable and reproducible ones (28). Another method is based on features' mathematical definitions. It makes *a priori* selection of features on those definitions and selects targeted parameters (29). In the formal process of analysis, different kinds of image analysis software may output a variety of features ranging from hundreds to thousands (30, 31). The inclusion of all features without selection in the development of radiomics model could cause the result in overfitting inevitably, since some of them might have a high degree of correlation (32). Hence, the inclusion of appropriate features, which are strongly linked to the aiming task but not redundant, is highlighted for improved value for specific clinical applications.

#### Modeling Methodology

ML is commonly used in radiomics model development, which can be hypothetically defined as a branch of artificial intelligence (AI) (33), which is actually an algorithm trained by inferences from data sets and then helps establish prediction models with high precision and efficiency on the basis of radiomic analysis. As a result, radiomics with ML may improve the clinician decision-making process as it is able to encompass many greater quantities of parameters than manual work and make these various parameters extracted in the workflow of radiomics into comprehensive utilization. With regard to the choice of appropriate radiomic methodology, the identification and application of optimal ML methods for radiomic applications are very crucial steps toward the achievement of clinical relevance. Thus, appropriate ML algorithms should be employed (34) to fuel the detective, diagnostic, and prognostic powers of radiomics in the field of bladder cancer.

#### Model Validation

Model validation is a dispensable step in model building, which serves as a useful tool to assess the performance and applicability of the developed radiomics model. To make sure that the model is effective for all of the targeted patients, not only the patients selected in the model building process, the internal and/or external validation should be tested. Typically, model performance is measured according to its discrimination, which can be expressed in the form of the receiver operating characteristic (ROC) curve or be calculated as the area under the ROC curve (AUC) in a quantitative way. ROC curve can easily display the ability of disease recognition at any threshold. When comparing two or more models, the ROC curves can draw each model in the same coordinate to identify advantages and disadvantages visually.

## Clinical Applications of Radiomics With ML in Bladder Cancer Management

### Accurate Cancer Staging and Grading

Accurate grading is of vital importance in the follow-up management of bladder cancer and serves as the starting point of radiomics applied in bladder cancer as shown in Table 1. Reading radiographic images produced by CT, MRI, PET, or US is essentially a matter of identifying complex patterns, in which computers can be trained to process efficiently, repeatedly, and rapidly. In the recent 5 years, ML techniques have shown latent capacity in accurate grading of bladder cancer. Confronted with the great significance of precise staging in the decision of appropriate treatment plan, Garapati et al. (35) developed a predictive model to serve as a classification tool for layering bladder cancer into two different grading categories and chose T2 as the critical point. They created a data set containing 84 bladder cancer lesions from 76 CT urography (CTU) cases and found that the morphological features, as well as texture features were helpful to stage the lesions of bladder cancer. In this study, the data were divided into two subsets for further two-fold cross validation. As a result, all of the linear discriminant analysis (LDA), neural network (NN), support vector machine (SVM), and random forest (RAF) classifiers included in this study led to relatively unanimous results in their staging accuracy, which effectively demonstrated that ML method can be a promising way to reduce the inaccuracy rate of bladder cancer staging by up to 50% (45) and aid to the implementation in daily care. To achieve better grading of bladder cancer for the sake of appropriate clinical decision, a recent study proposed textural features from diffusion-weighted imaging (DWI) and apparent diffusion coefficient (ADC) maps to distinguish low-grade bladder cancers from high-grade ones and recommended an optimal feature subset selected by SVM with recursive feature elimination (SVM-RFE) for cancer grading using histogram and gray-level co-occurrence matrix (GLCM)-based radiomic features (36). Sixty-one patients with bladder cancer were included in this study to prove that the grading performance in bladder cancer was improved through the candidate or extraction of optimal features, with accuracy, sensitivity, specificity, and AUC achieving 82.9, 78.4, 87.1, and 0.861, respectively.

**Table 1:** Present studies that combined radiomics and machine learning (ML) in bladder cancer.

**References**	**Study type**	**Application**	**Cases number**	**Data modality**	**ML algorithm**	**Type of validation**	**Results**
Garapati et al. (35)	Retrospective study	Bladder cancer staging	76	CTU	LDA, NN, SVM, RAF	Two-fold cross validation	Four types of classifier showed equal promise in bladder cancer staging
Zhang et al. (36)	Retrospective study	Bladder cancer grading	61	MRI	SVM-RFE	Single-center validation	The SVM classifier adapting the optimal feature subset performed best (AUC = 0.861; accuracy 82.9%; sensitivity 78.4%; specificity 87.1%)
Wang et al. (37)	Retrospective study	Bladder cancer grading	70	MRI	LASSO algorithm	Ten-fold cross validation	Joint-Model performed best (AUC = 0.9276)
Zheng et al. (38)	Retrospective study	Differentiation of NMIBC and MIBC	199	MRI	LASSO logistic regression algorithm	Single-center validation	The radiomic-clinical nomogram developed on the basis of three-dimensional features showed favorable usage (AUC 0.922)
Wang et al. (39)	Retrospective study	Prediction of mortality after radical cystectomy	117	Clinical data	BPN, RBFN, ELM, RELM, SVM, NB, and KNN	Ten-fold cross validation	The models with RELM and ELM achieved the highest sensitivity and specificity (over 0.8)
Xu et al. (40)	Retrospective study	Recurrence stratification of bladder cancer	71	MRI	SVM-RFE, LASSO algorithm	Five-fold cross validation	The radiomic clinical nomogram achieved more benefits than the radiomics or clinical model alone
Lin et al. (41)	Retrospective study	Prediction of progression-free interval	62	CECT	LASSO algorithm	Single-center validation	Radiomics risk model (AUC 0.956) and transcriptomics risk model (AUC 0.948) showed independent prognostic role to determine the progression
Cha et al. (42)	Retrospective study	Assessment of therapy response	62	CT	DL-CNN	Leave-one-case-out cross validation	DL-CNN has the potential to assist in the treatment response
Cha et al. (43)	Retrospective study	Assessment of treatment response	123	CT	DL-CNN	Single-center validation	The radiomics-based system is advisable to serve as a second option to assist in therapy evaluation
Chalkidou et al. (44)	Retrospective study	Evaluation of sensitivity to neoadjuvant chemotherapy	123	CT	DL-CNN	Single-center validation	The improvement of the physicians' performance was statistically significant (P <.05)

*LDA, linear discriminant analysis; NN, neural network; DWI, diffusion-weighted imaging; ADC, apparent diffusion coefficient; RFE, recursive feature elimination; BPN, back-propagation neural network; RBFN, radial basis function; ELM, extreme learning machine; RELM, regularized ELM; NB, naive Bayes; KNN, k-nearest neighbor; DL-CNN, deep-learning convolution neural network; MIBC, muscle-invasive bladder cancer; NMIBC, non-muscle-invasive bladder cancer*.

Recently, there have also been notable advances in MRI technology. Wang et al. (37) built and validated a multiparametric MRI-based radiomic analysis model for the preoperative grading of bladder cancer tumors. This study enrolled 70 bladder cancer patients and applied five radiomic models including T2-weighted imaging (T2WI), DWI, ADC, Max-out, and Joint models, then assessed by ROC curve analysis. By comparing AUC values, the performance in terms of the accuracy, sensitivity, and specificity of the Joint_model in the validation set was obviously superior to that of the other four single-modality models, achieving an AUC of 0.9233 according to the training cohort and 0.9276 in the validation one. As the first study considering pathological grading of bladder cancer applying radiomics, it showed encouraging feasibility for avoiding subjectivity and promote extended future usage in preoperative grade assessment of bladder cancer.

### Tumor Classification and Prognosis Prediction

Undoubtedly, clear discrimination between NMIBC and MIBC is crucial for pretreatment decision, posttreatment prognosis, and lasting period consequent management of bladder cancer patients. Considering the large percentage of diagnostic errors caused by conventional cystoscopic examination (36), researches proposed a new radiomics scheme that combined histogram features, co-occurrence matrix (CM) features, and run-length matrix (RLM) features. Based on the novel developed model (4), they assessed the performance of tumor classification based on multiparametric MRI radiomic features for accurate differentiation between NMIBC and MIBC preoperatively in searching for management of bladder cancer patients and finally got a positive result with the AUC and Youden index improving to 0.8610 and 0.7192, respectively.

With regard to the reflection on the heterogeneity of cancer caused by analyzing radiomic features extracted from the whole tumor tissue, Zheng et al. (38) suggested a hypothesis that the radiomics features of the basal part can be used for determining the degree of muscle invasiveness more conclusively. To further validate their assumption, they first developed a radiomic-clinical nomogram incorporating the radiomic signature and extract three-dimensional features for subsequent analysis. The usefulness of this novel nomogram in discriminating NMIBC and MIBC was favored, with an AUC of 0.922 in the training set, and was also confirmed with an AUC of 0.876 in the validation set. By overthrowing existing views, this study came up with a new idea in the extraction of radiomic features and demonstrated the latent capacity of the radiomic-clinical nomogram to serve as an auxiliary tool for bladder cancer classification.

Realizing the high risk of metastasis and mortality of MIBC that is in need of immediate treatment to improve the living quality of patients while faced with the various kinds of ML models emerging, researchers have begun to seek for the urgently needed strategy. A confirmative research was conducted for selection in which seven models including radial basis function (RBFN), back-propagation neural network (BPN), extreme learning machine (ELM), regularized ELM (RELM), SVM, naive Bayes (NB) classifier, and k-nearest neighbor (KNN) were ever explored and compared to predict the 5-year mortality of 117 MIBC patients who had undergone RC (39). The eventual results indicated that the algorithm on the basis of RELM and ELM presented higher mean sensitivity and specificity (over 0.8) relatively in the prediction of mortality in MIBC patients after RC, with an ideally fast learning speed.

Regarding the importance of preoperative prediction of the risk of bladder cancer recurrence, Xu et al. (40) developed a radiomic nomogram for personalized prediction of the first 2 years (TFTY) risk in tumor recurrence. This study took the important baseline variations involving gender, age, cancer grading, MIS of the lesions, size, and the number of the tumor, as well as the recording of previous operations into consideration to ensure the accuracy of the developed model. This multiparametric model finally showed excellent performance in both the validation and training cohorts. The decisive curve exhibited the threshold of risk was more than 0.3; more benefits and higher accuracy were observed by applying the radiomic nomogram than using either the radiomics or clinical model separately.

All of these studies showed the great potential of radiomics aided by ML in the improvement of tumor classification and prognosis prediction of bladder cancer, overcoming the defects traditionally and obviously improved the living quality of bladder cancer patients. Great progression as radiomics made in the prognosis of bladder cancer, the correlations between the imaging features and genomic signatures have rarely been explored up to now in the field of bladder cancer management. Confronting the blank of related researches, Lin et al. (41) put forward a successful case that integrated radiomics and transcriptomics to predict the progression-free interval (PFI) of bladder cancer patients. In this study, both of the radiomics risk model (AUC 0.956) and transcriptomics risk model (AUC 0.948) showed independent prognostic roles to determine the progression of the bladder tumor, which first provided a novel insight into the microscopic mechanisms of bladder cancer.

### Evaluation of Individual Therapy Responses

Once a definite diagnosis is made and the tumor subtype is identified, early evaluation of therapeutic efficacy and response can aid in clinicians'decision on whether to discontinue chemotherapy at an optimal phase. In the research conducted by Cha et al. (42), the feasibility of a radiomics-based prognostic model using CT images obtained before or after treatment in distinguishing bladder cancers with chemotherapy responses or not was explored, which applied deep-learning convolution neural network (DL-CNN) for accurate bladder lesion segmentation. This study indicated that the computerized assessment on the basis of radiomics information extracted from CT images of pretreatment or posttreatment bladder cancer patients had the possibility to aid in the evaluation of therapy response, with the prediction accuracy estimated by AUCs improving by 0.03 in comparison with manual contours. Previously, one of the reasons for optimization has been demonstrated that the complicated tumor size change in response to treatment can be better reflected by the computerized three-dimensional (3-D) segmentation rather than traditional criteria (46). Recognizing the important role played by radiomics and ML in the evaluation of individual therapy response, another feasibility study adopted three radiomics predictive models in the assessment of chemotherapy response and finally reached an agreement among all these models and two expert radiologists' prediction. Instead of replacing artificial analysis, researchers are more supportive to consider the computer-aided system as a second option to assist in the evaluation process. However, the final decision still relies on the judgment of radiologists on the basis of advice given by radiomics-aided models (43).

Regarding the aggressiveness of MIBC, studies were conducted to assess whether a CT-based decision-support system could improve identification of patients who have complete or partial response to neoadjuvant chemotherapy (47). This study investigated 123 subjects, and the AUC was estimated. Compared to the accuracy of the assessment of doctors alone (AUC 0.74), the decision-support system (AUC 0.80) showed statistically significant improvement of therapeutic evaluation.

## Challenges and Future Directions

Up to now, there has been a large number of studies to illustrate the rapid development of radiomics and ML algorithms, as well as the effectiveness of their combination in the full-cycle management of bladder cancer, but we also need to realize the limitations when applied in actual use comprehensively.

First, the defects in the aspect of inherent technologies should be recognized. Usefulness as quantitative features based on medical images show in the management of bladder cancer patients, their tendency of causing significant errors and overestimation has also been reported (44). It is prevalent to see that the number of radiological features examined is much larger than the number of patients included in retrospective studies, which may lead to bias in feature selection and even high false-positive result. Furthermore, results obtained from different studies might be hard to compare and evaluate because of the lack of standardized methods for analysis. Larue et al. (48) conducted an overview and put forward a thought-provoking conclusion that there still exist various kinds of challenges to overcome in the whole process of radiomics. In terms of ML, although specific algorithms can improve the accuracy of the prediction with the usage of regression models by extracting complex features in the data set, no amount of algorithm skills or computing power can extrude unwanted information (49).

Second, limitations mentioned in current articles that have already been published should be highlighted and overcome. Quite a large percent of studies are conducted in a single institution, and the sample size is too small to be convincing. Apart from that, some studies lack external validation for model development. For the extensive clinical application, the studies should be designed in a much more comprehensive and delicate way to raise the reliability of research results. To achieve future optimization of this novel method, more investigations should be carried out to test the potential of optimizing the predictive model by the combination of imaging biomarkers with other non-imaging biomarkers.

Third, it has been a long-lasting debate whether such AI-supported systems are much smarter than clinical practitioners (50, 51). However, what we should do is to take the best advantages of the new adjuvant imaging techniques and apply radiomics together with ML to bladder cancer monitoring. Combining all of these imaging analysis methods with the experience of experts will make assurance for the delivery of medical care that outperforms what either of them can achieve alone. Since no code is shared in most papers involving radiomics application, it is impossible to exactly assess the validity of the results up to now. Thus, another direction for future improvement is to achieve the validity of predictive results based on the public full code and imaging data sets and optimize the model performance in a more scientific way.

Moreover, the application of radiomics in bladder cancer remains at the macroscopic level. Although we have witnessed the breakthrough of various researches from basic tumor detection to accurate grading, even recent studies have moved toward the prediction of treatment outcomes. However, gene therapy and immunotherapy, which involve gene expression signature and immune phenotype, have been introduced as revolutionary tools for comprehensive tumor assessment and divert our attention to the microscopic level. A novel concept named “radiogenomics” perfectly presents the integration of genomics with radiomics and serves as an alternative to the invasive biopsy (52). Tremendous value as this research spot shows in several common diseases (53, 54), few studies are conducted to further validate its clinical uses in bladder cancer, which highlights that the combination of radiogenomics and bladder cancer full-cycle management can be an essential breakthrough point for future research.

## Author Contributions

LG and YC collected and reviewed the literature, and wrote the manuscript. JL helped with the writing design and revised the manuscript. PZhao, PZhan, and RA provided helpful comments on the manuscript. All authors read and approved the final manuscript.

### Conflict of Interest

The authors declare that the research was conducted in the absence of any commercial or financial relationships that could be construed as a potential conflict of interest.
